# Integrated analysis of next generation sequencing minimal residual disease (MRD) and PET scan in transplant eligible myeloma patients

**DOI:** 10.1038/s41408-023-00794-x

**Published:** 2023-03-06

**Authors:** Rodrigo Fonseca, Mariano Arribas, Julia E. Wiedmeier-Nutor, Yael N. Kusne, Miguel González Vélez, Heidi E. Kosiorek, Richard (Duke) J. Butterfield, Ilan R. Kirsch, Joseph R. Mikhael, A. Keith Stewart, Craig Reeder, Jeremy Larsen, P. Leif Bergsagel, Rafael Fonseca

**Affiliations:** 1 Division of Hematology and Medical Oncology, Mayo Clinic, Phoenix, AZ USA; 2 Dignity Health Cancer Institute, Phoenix, AZ USA; 3 Department of Health Sciences Research, Mayo Clinic, Scottsdale, AZ USA; 4 Translational Medicine, Adaptive Biotechnologies, Seattle, WA USA; 5 Translational Genomics Research Institute, City of Hope Cancer Center, Phoenix, AZ USA; 6 Princess Margaret Cancer Centre, University Health Network, Toronto, ON Canada

**Keywords:** Translational research, Myeloma, Myeloma

## Abstract

Minimal residual disease (MRD) assays allow response assessment in patients with multiple myeloma (MM), and negativity is associated with improved survival outcomes. The role of highly sensitive next generation sequencing (NGS) MRD in combination with functional imaging remains to be validated. We performed a retrospective analysis on MM patients who underwent frontline autologous stem cell transplant (ASCT). Patients were evaluated at day 100 post-ASCT with NGS-MRD and positron emission tomography (PET-CT). Patients with ≥ 2 MRD measurements were included in a secondary analysis for sequential measurements. 186 patients were included. At day 100, 45 (24.2%) patients achieved MRD negativity at a sensitivity threshold of 10^−6^. MRD negativity was the most predictive factor for longer time to next treatment (TTNT). Negativity rates did not differ according to MM subtype, R-ISS Stage nor cytogenetic risk. PET-CT and MRD had poor agreement, with high rates of PET-CT negativity in MRD-positive patients. Patients with sustained MRD negativity had longer TTNT, regardless of baseline risk characteristics. Our results show that the ability to measure deeper and sustainable responses distinguishes patients with better outcomes. Achieving MRD negativity was the strongest prognostic marker and could help guide therapy-related decisions and serve as a response marker for clinical trials.

## Introduction

The development of new regimens, in combination with autologous stem cell transplant (ASCT), have resulted in unprecedented rates of complete response (CR) and improved overall survival (OS) in patients with multiple myeloma (MM) [1–8]. Although, patients that achieve CR have a prolonged progression-free survival (PFS), a significant proportion of patients achieving CR after first line therapy eventually relapse. Relapses after achieving CR are likely secondary to disease persistence below the limit of detection of traditional MM laboratory markers [3, 9, 10]. The International Myeloma Working Group (IMWG) criteria were updated in 2016 to further classify patients who achieve a CR utilizing minimal (or measurable) residual disease (MRD) and functional imaging [3, 11–14]. MRD is a marker of disease that can be determined using either next-generation flow (NGF) or next-generation sequencing (NGS) and is now standard of care for other hematologic malignancies [15–22].

Recent meta-analyses have shown that achieving MRD negativity is associated with significant improvement in PFS and OS in transplant eligible, transplant ineligible, and relapsed/refractory disease patients with MM [23, 24]. This effect on prognosis and survival is observed regardless of treatment type and cytogenetic risk [18, 24–27]. When adjusted for other variables, including cytogenetic risk and depth of clinical response, MRD is the strongest prognostic factor for PFS, and the benefit of attaining CR loses independent significance [25–28]. Deeper responses, which are apparent with increased sensitivity of MRD techniques, further improve outcome [23, 24, 26, 29–31]. Although MRD negativity is associated with improved survival in all thresholds for patients with MM, outcomes for PFS and OS were greatest when patients reach MRD negativity at a sensitivity of 10^−6^ (PFS: HR 0.22, 95% CI 0.16–0.29, *p* < 0.001 and HR 0.38, 95% CI 0.32–45, *p* < 0.001 for sensitivity to 10^−6^ and 10^-^^4^ respectively; OS: HR 0.26, 95% CI 0.13–0.51, *p* < 0.001 and HR 0.50, 95% CI 0.43–0.60, *p* < 0.001 respectively) [24]. It has been shown that there is approximately a 1-year survival benefit for each 1-log depletion in tumor burden in patients with MM [27]. MRD could also serve as a highly relevant and useful measure of response for MM clinical trials in an era where there is increasing complexity of treatment schedules and improving rates of CR [32].

Very few studies have evaluated the effect on prognosis by evaluating MRD to a sensitivity of 10^−6^, and only three of these studies used next generation sequencing [19, 20, 26, 33, 34]. This study seeks to determine the utility of MRD outside the context of clinical trials, and the complementary roles of functional imaging and sequential MRD measurements. We examined a large cohort of transplant eligible patients, with multiple treatment regimens and risk groups, who have undergone MRD evaluation at day 100 post-ASCT with NGS at a sensitivity of at least 10^−6^.

## Methods

This study was approved by the Institutional Review Board. We performed a retrospective analysis on a cohort of patients diagnosed with MM between January 2015 and August 2020. We included patients who received frontline ASCT, regardless of induction regimen, and then underwent bone marrow evaluation 100 days after ASCT ( + /− 10 days). All patients received frontline induction therapy followed by a single ASCT with high dose melphalan conditioning. Patients who received multiple lines of therapy prior to ASCT or who had tandem ASCT, but with no MRD evaluation after first transplant were excluded from the study.

Response was evaluated at day 100 post-ASCT according to IMWG criteria, and subclassified based on functional imaging and MRD evaluation. MRD analysis was measured using the FDA cleared NGS clonoSEQ® Assay (Adaptive Biotechnologies Corporation, Seattle, USA), with a sensitivity of < 10^−6^ depending on the total number of nucleated cells’ worth of DNA assessed [35]. Briefly, the assay tracks and quantifies disease-associated immunoglobulin gene sequence rearrangements, identified as “dominant” in a bone marrow sample at time of diagnosis. Patients with two or more MRD measurements, taken at least 6 months apart, were included in the sequential MRD analysis. In order to reduce bias in patients with more than two measurements, only the first two measurements taken a year apart were included in the sustained MRD negativity analysis.

Imaging evaluation was performed using PET/CT scan at same time of bone marrow evaluation, using a Gemini GXL10 scanner (Philips Medical Systems) and interpreted by certified radiologists [12]. Clinical information, disease features and patient characteristics were obtained through chart review of electronic clinical charts. Detailed annotations related to consolidation and/or maintenance therapy post-ASCT were also collected.

Disease risk was classified in our population according to the Revised International Staging System (R-ISS) [36]. If not documented in patient record, R-ISS score was calculated using baseline data, if available. Cytogenetic risk was based on the IMWG molecular classification [37]. An additional category was created in an attempt to obtain a more profound understanding of the role of chromosome 1q21. Patients with standard risk features who presented two or more extra copies (4 or more total copies) of 1q21 (amplification of 1q) were labeled as “High Risk Plus”, while those who presented one extra copy (3 total copies, gain 1q) were kept as “Standard Risk” [37, 38]. We used this novel category for statistical analysis.

Assessment of progression free survival (PFS) is challenging in retrospective research due to the lack of consistent follow-up intervals. Therefore, we used time to next treatment (TTNT), regardless of patient response after induction therapy plus ASCT [39]. We defined TTNT as the time from ASCT until the start of a new line of therapy driven by disease progression. Consolidation and maintenance therapy were not considered events for TTNT. Patients in whom therapy was changed or adjusted due to side effects also were not considered events for TTNT. Overall survival (OS) was defined as the time from diagnosis until death. Patients without a TTNT or death event were considered censored. Patient data was last revised and updated on February 9th, 2022

Normality tests were performed and association testing for categorical variables was done using Chi-Squared test. Testing for continuous variables used either a Student t-test or an analysis of variance (ANOVA). MRD positivity values were log-transformed for analysis. Survival distributions were estimated using the Kaplan-Meier method and compared between groups by the log-rank test. The prognostic value of MRD was evaluated using univariate and multivariable Cox proportional hazard models. TTNT was evaluated separately for 10^−4^, 10^−5^ and 10^−6^ sensitivity groups. Agreement between MRD sensitivity and PET-CT interpretation was evaluated using the kappa statistic in patients who had PET-CT done. Statistical analysis was done using SAS version 9.4 (SAS Institute, Cary, NC) and GraphPad Prism 9 software. All tests were 2-sided and a *p*-value of < 0.05 was used for statistical significance.

## Results

A total of 353 patients with a diagnosis of MM underwent MRD assessment. Excluded from the analysis were 141 patients who were not treated with ASCT and 26 patients who did not undergo ASCT as frontline therapy. The remaining 186 patients were included in the analysis (Supplementary Fig. 1). Twenty-six patients did not undergo ASCT as frontline therapy and were excluded from the analysis. Demographic characteristics are summarized in Table 1. On average, patients were evaluated 92 days after ASCT, with a median time of follow up of 39.8 months from initial diagnosis. The median age at diagnosis was 62.5 years. Eighteen (11.3%) patients were R-ISS Stage III, and based on our genetic risk classification, 34 patients (19.2%) had high-risk cytogenetics. At day 100, 119 (64.0%) patients had achieved CR or better, 48 (25.8%) achieved very good partial response (VGPR), 16 (8.6%) partial response (PR), 1 (0.5%) minimal response and 2 (1.1%) had progressive disease.

**Table 1:** Patient demographic characteristics and response status at day 100 Post-Autologous Stem Cell Transplant (ASCT).

Overall	Overall (*N* = 186)
**Gender**	
Female	65 (34.9%)
Male	121 (65.1%)
**AGE**	
Mean (SD)	60.495 (8.731)
Median	62.5
Range	36.0, 75.0
**R-ISS Stage**	
I	68 (42.8%)
II	73 (45.9%)
III	18 (11.3%)
*Missing*	27
**High-risk cytogenetics**	
No	150 (85.7%)
Yes	25 (14.3%)
*Missing*	11
**High-risk “Plus” Cytogenetics**	
No	143 (80.8%)
Yes	34 (19.2%)
*Missing*	11
**IMWG Response criteria (at day 100)**	
Stringent Complete Response	101 (54.3%)
Complete Response	18 (9.7%)
Very Good Partial Response	48 (25.8%)
Partial Response	16 (8.6%)
Minimal Response	1 (0.5%)
Progressive Disease	2 (1.1%)
**Binary response criteria**	
Complete Response	119 (64%)
Non-Complete Response	67 (36%)
**Pet Interpretation (at day 100)**	
Negative	111 (81.6%)
Positive	25 (18.4%)
*Missing*	50
**MRD Negativity by sensitivity**	
Positive	70 (37.6%)
10^−^^4^	33 (17.7%)
10^−5^	38 (20.4%)
10^−^^6^	45 (24.2%)
**10**^**−**^^**6**^ **Sensitivity threshold**	
Negative	45 (24.2%)
Positive	141 (75.8%)
**10**^**−5**^ **Sensitivity threshold**	
Negative	82 (44.1%)
Positive	104 (55.9%)
**Consolidation therapy**	
No	152 (81.7%)
Yes	34 (18.3%)
**Consolidation regimen**	
DRd	13 (38.2%)
IRd	6 (17.6%)
KRd	6 (17.6%)
Other	9 (26.6%)
**Maintenance therapy**	
No	30 (16.1%)
Yes	156 (83.9%)
**Maintenance regimen**	
PI Monotherapy	8 (5.1%)
PI Combination	2 (1.3%)
IMID-PI Combination	26 (16.7%)
IMID Monotherapy	101 (64.7%)
IMID Combination	13 (8.3%)
Other	6 (3.9%)

*DRd* Daratumumab, Revlimid, Dexamethasone; *IRd* Ixazomib, Revlimid, Dexamethasone; *KRd* Kyprolis, Revlimid, dexamethasone, *PI* Proteosome inhibitor, *IMID* Immunomodulatory agent.

Forty-five (24.2%) patients of the total population achieved MRD negativity at a sensitivity threshold of 10^−6^. An additional 38 (20.4%) patients had disease being between 10^−6^ and 10^−5^ log, and consequently would have been negative with a less sensitive test using a threshold of 10^-5^. Interestingly, 5 patients (11.1%) who had achieved VGPR, achieved MRD negativity at 10^−6^. At the time of this writing, only one has had disease progression and another had a non-disease associated death (with no registered progression). None of the patients with PR, MR or PD achieved negativity. Of the 45 patients negative at a sensitivity of 10^−6^, 14 had disease detectable below the limit of detection (LOD) or limit of quantification (LOQ), (refer to Adaptive Biotechnologies clonoSEQ® Assay technical information for additional details). Following ASCT, 136 (73%) patients received maintenance therapy alone, 20 (11%) patients received both consolidation and maintenance therapy and 14 (7%) patients received only consolidation. The majority (89.7%) of maintenance regimens contained IMIDs, whether alone or in combination with other agents.

**Fig. 1:**
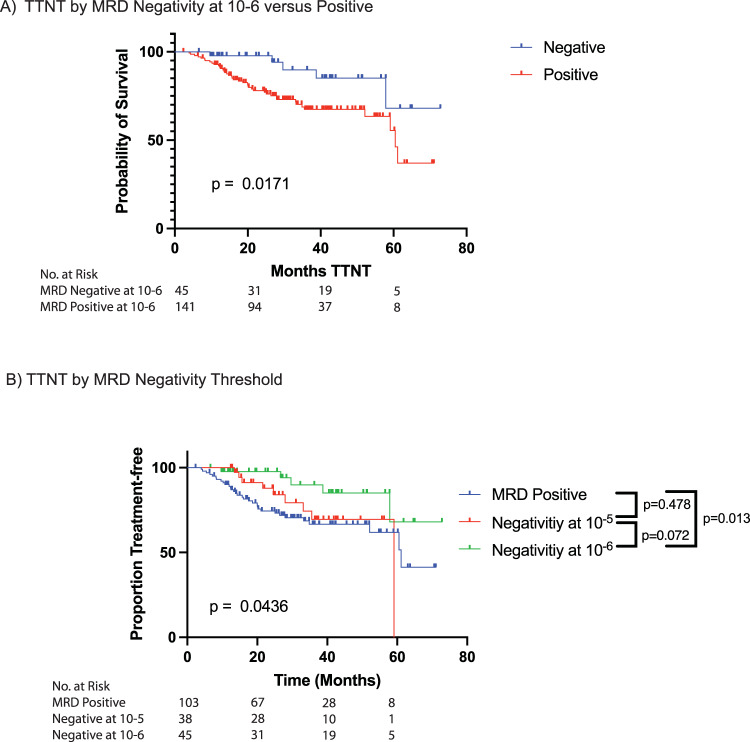
Kaplan Meier curves for time to next treatment (TTNT) based on MRD status. Based on MRD status at Day 100 Post-ASCT evaluation. **A** The median TTNT was not reached for those patients who achieved negativity at 10^−^^6^; and was 60.5 months for patients who remained positive. **B** The median TTNT was not reached for reached for patients who achieved MRD negativity at 10-6, 59.1 months in those who achieved negativity at 10^−^^5^, and 61 months who remained MRD positive.

At cutoff, the follow up median time after ASCT was 27.5 months and 46 (24.7%) patients had progressed based on TTNT criteria. Of those who progressed, 27 (59%) had obtained CR or stringent CR (sCR) at day 100 evaluation. In a univariate model, only MRD negativity at 10^−6^ was associated with better TTNT (HR: 0.289, 95% CI: 0.110–0.758, *p* = 0.01, Table 2). Patients achieving negativity at 10^−6^ had longer TTNT, even when compared to patients achieving negativity at 10^−5^ (Fig. 1). Therefore, this threshold was used to define MRD negativity for further analysis.

**Table 2:** Cox proportional hazard model for Time to Next Treatment (TTNT) and Overall Survival (OS) by Minimal Residual Disease (MRD) levels at day 100 Post-Autologous Stem Cell Transplant (ASCT).

	Time to Next Treatment (TTNT)			Overall Survival (OS)		
MRD level at Day 100 post-ASCT	Adjusted Hazard Ratio	95% Confidence Interval	*P*-value	Adjusted Hazard Ratio	95% Confidence Interval	*P*-value
[10^−5^–10^−^^4^] vs >10^−4^	0.699	0.312–1.563	0.38	0.938	0.240–3.668	0.93
[10^−6^–10^−^^5^] vs >10^−4^	0.692	0.319–1.502	0.35	1.237	0.355–4.316	0.74
< 10^−6^ vs > 10^−4^	**0.289**	**0.110**–**0.758**	0.01*	0.619	0.160–2.401	0.49

Bold values identify statistical significance (*p* < 0.05)

On multivariable analysis, adjusting for R-ISS stage, IMWG response category, cytogenetic risk status, and MRD negativity, achieving negativity at 10^−6^ was the strongest prognostic factor for longer TTNT (HR = 0.35, 95% CI 0.12–1.03, *p* = 0.06; Table 3). There were no significant differences in TTNT nor OS between those who achieved true negativity at 10^−6^ and those who had disease detectable below LOD or LOQ (Supplementary Fig. S2). However, given the post hoc nature of our analysis and limited dataset size, we cannot exclude possible differences in survival outcomes.

**Table 3:** Univariate and Multivariable Cox Proportional Hazard Model for Time to Next Treatment (TTNT) and Overall Survival (OS).

		Time to Next Treatment (TTNT)				Overall Survival (OS)			
		Univariate		Multivariable		Univariate		Multivariable	
Variable	Level	HR (95% CI)	*P*-Value	HR (95%)	*P*-value	HR (95% CI)	*P*-Value	HR (95%)	*P*-value
MRD negativity sensitivity 10^−6^	Negative vs. Positive	**0.34 (0.13**–**0.86)**	0.02*	0.35 (0.12–1.02)	0.06	0.60 (0.17–2.09)	0.42	0.55 (0.12–2.55)	0.44
Genetic Risk	High Risk vs. Low Risk	**2.46 (1.34**–**4.52)**	0.004 *	1.96 (0.94–4.11)	0.07	**4.01 (1.5**–**10.71)**	0.006 *	1.12 (0.33–3.78)	0.86
IMWG Response	CR or better vs. Not	0.77 (0.42–1.38)	0.38	0.79 (0.41–1.50)	0.47	1.05 (0.39–2.83)	0.93	1.06 (0.33–3.43)	0.92
R-ISS Stage	Stage II vs. Stage I	1.38 (0.68–2.80)	0.37	1.09 (0.52–2.30)	0.81	2.61 (0.53–12.98)	0.24	1.94 (0.36–10.55)	0.44
	Stage III vs. Stage I	**3.63 (1.53**–**8.62)**	0.004 *	2.46 (0.93–6.47)	0.07	**26.17 (4.98**–**137.7)**	<0.001 *	**25.226 (4.02**–**158.40)**	<0.001*

Bold values identify statistical significance (*p* < 0.05)

As for OS, there were a total of 17 (9.1%) registered deaths. MRD alone did not predict OS (Supplementary Fig. S3). In the multivariable model, only the presence of R-ISS Stage III was associated with worse outcome (Table 3; Supplementary Fig. S4). MRD predicted worse OS only in the context of combining it with cytogenetic risk (positive MRD and presence of high-risk cytogenetics; HR: 9.74, 95% CI: 1.19–79.51, *p* = 0.04). This may be due to the relative limited time of follow up and low number of deaths in our cohort. Survival analyses limited only to patients who achieved CR or sCR at day 100 (*n* = 120) showed similar results (Supplementary Table S1).

**Fig. 2:**
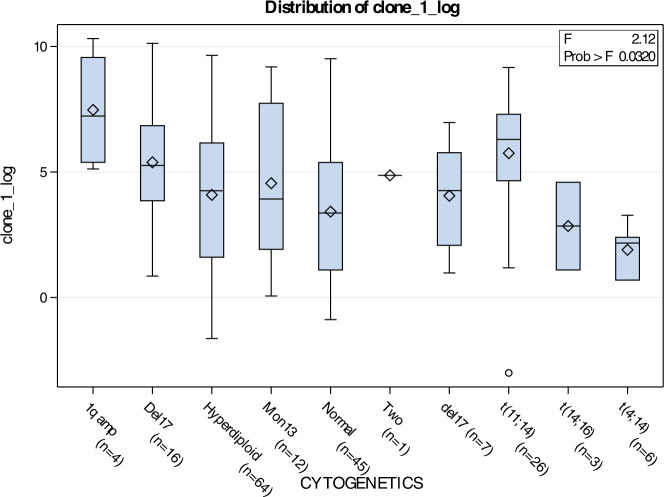
Distribution of MRD clone by cytogenetic event. Log transformed MRD clone MRD and patients categorized based on primary cytogenetic events. General linear model (GLM) shows statistically higher levels in patients with 1amp (4 or more copies).

MRD negativity rate did not differ according to MM subtype, R-ISS stage, or cytogenetic risk group (Supplementary Table S2). When MRD was treated as a continuous variable (log-transformed), only positive PET-CT interpretation and response lower than sCR were associated with higher clone levels (Table 4). We also compared MRD levels between patients with common MM cytogenetic events and found that 1q amplification was associated with higher MRD values (number of residual clonal cells per million nucleated cells) compared to others (in cases where MRD remained positive at Day 100 Post-ASCT; Fig. 2), although there was a small number of patients with this cytogenetic event.

**Table 4:** Minimal Residual Disease (MRD) Positivity Value (Clone 1 – Log) by Baseline Disease Characteristics and IMWG Response Criteria at Day 100 Post-Autologous Stem Cell Transplant (ASCT).

CATEGORY	MEAN (SD)	MEDIAN	*P*-value
**GENDER**			0.531
Female	4.0 (2.95)	3.4	
Male	4.3 (2.92)	4.6	
**MM Subtype**			0.731
IgA Kappa	3.9 (2.74)	3.3	
IgA Lambda	4.4 (2.81)	4.1	
IgD Lambda	3.9 (NA)	3.9	
IgG Kappa	4.4 (2.93)	4.7	
IgG Lambda	4.7 (2.75)	5.1	
IgM Kappa	5.3 (NA)	5.3	
Kappa Light Chain	3.6 (3.27)	3.3	
Lambda Light Chain	3.6 (3.49)	1.7	
Non-Secretory	0.8 (5.32)	0.8	
**R-ISS Stage**			0.131
Stage I	4.2 (2.73)	4.7	
Stage II	4.6 (3.16)	4.4	
Stage III	2.9 (2.81)	2	
**Genetic risk**			0.531
High Risk	3.8 (2.61)	3.3	
Standard Risk	4.3 (3.01)	4.5	
**Modified genetic risk**			0.841
High Risk “Plus”	4.3 (3.01)	4.6	
Standard Risk	4.2 (2.94)	4.3	
**Pet interpretation**			0.002^1^*
Positive	5.7 (2.58)	5.8	
Negative	3.7 (2.89)	3.6	
**IMWG Response criteria**			<0.001^1^*
sCR	2.2 (2.16)	1.9	
CR	5.1 (2.70)	5.3	
VGPR	5.7 (2.14)	5.7	
PR	7.2 (1.94)	7.4	
MR	8.6 (NA)	8.6	
PD	7.5 (3.72)	7.5	

^1^ANOVA F-test *p*-value

*Statistically significant *p*-value

At day 100, 136 patients had PET-CT interpretation results. Agreement between MRD and PET-CT interpretation was poor (Kappa = 0.10, 95% CI 0.02-0.17; Supplementary Table S3), primarily due to PET-CT being negative in MRD positive patients. Out of the 102 patients who had positive MRD, only 23 (22.6%) had positive PET-CT. Two patients had positive PET-CT and negative MRD assay, but neither had disease progression at the time of this writing, with follow-up times of 33.8 months and 19.7 months, respectively.

**Fig. 3:**
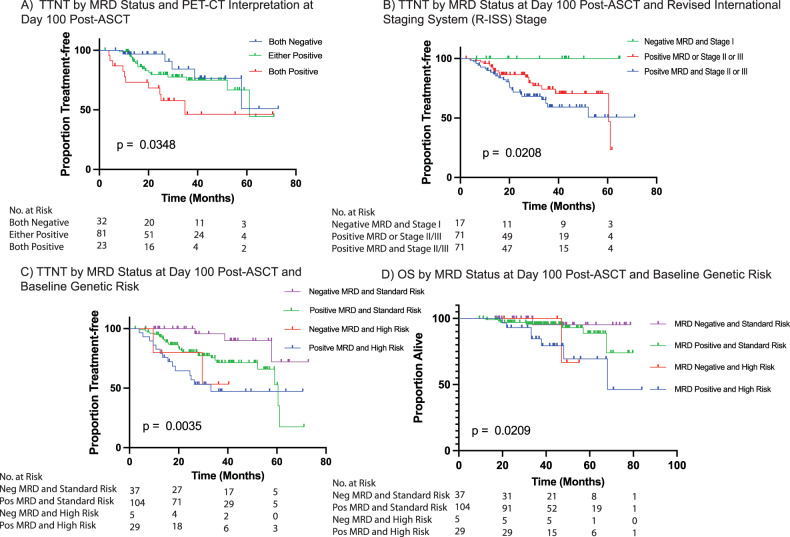
Kaplan Meier curves for time to next treatment (TTNT) and Overall Survival (OS) According to MRD Status in Combination with Other Disease Parameters. **A** According to MRD status and PET-CT Interpretation at day 100. Patients with both MRD and PET-CT negative did not reach median TTNT. Patients who had either MRD positive or PET-CT positive had a median TTNT of 61 months; this group was mostly compromised of patients with positive MRD and negative PET-CT (79; 97%) while only a small number of patients (2; 3%) were PET-CT positive/MRD-negative. Patients who had both assessment positive had a median TTNT of 34.9 months. **B** Based on MRD status at day 100, and R-ISS stage at time of diagnosis. Patients with MRD negativity and R-ISS Stage I did not reach a median TTNT. Patients who had both positive MRD and Stage II or Stage III did not reach a median TTNT either. Patients with either a positive MRD or Stage II or Stage III disease had a median TTNT of 60.5 months. **C** According to MRD status at day 100 and genetic risk at time of diagnosis. Patients with negative MRD and standard risk genetics, and patients with negative MRD and high-risk cytogenetics did not reach median TTNT. Those with positive MRD and standard cytogenetics had a median TTNT of 60.5 months, while those with positive MRD and high-risk cytogenetics had a median TTNT of 33.1 months. **D** OS according to MRD assessment at day 100 and genetic risk at time of diagnosis. Only patients with positive MRD and high-risk genetics reached a median OS of 68.1 months. The other three subgroups did not reach median OS.

Patients with both negative MRD and negative PET-CT at day 100 had significantly longer TTNT (Fig. 3A; Median TTNT: not reached in both negative, 61 months for either positive, 35 months for both positive; *p* = 0.03). As highlighted above, the majority of patients in the either positive group were patients with positive MRD and negative PET-CT (79; 97%). Combination of MRD and R-ISS stage was also associated with prognosis; those with Stage I and MRD negativity at day 100 had increased TTNT (Fig. 3B). Finally, MRD status in combination with cytogenetic risk can also predict outcomes, as those classified as low risk (MRD negative and standard risk cytogenetics) had better TTNT and OS as well (Figs. 3C and 3D).

A total of 57 patients had two MRD measurements taken at least 6 months apart, of which 54 (94.7%) received either consolidation therapy (6; 11%), maintenance therapy (36; 67%), or both (12; 22%). Additional therapy, whether consolidation or maintenance, was associated with increased rates of MRD negativity throughout (Supplementary Tables S4 and S5). The rate of MRD negativity at 10^−6^ improved from 24.4% (11/45) to 55.6% (25/25; *p* = 0.001) after at least 12-months of therapy; and from 28.6% (6/21) to 57.1% (12/21; *p* = 0.041) at time of completion of maintenance and/or consolidation therapy, at which point patients remained off therapy. (Supplementary Table S5 B2 and B3). Achieving MRD negativity, either at day 100 post-ASCT or until completion of consolidation/maintenance therapy, was associated with longer TTNT (Supplementary Figure S5). Patients had similar outcomes, regardless of if they attained MRD negativity at day 100 or later. The three (5.3%) patients that did not receive consolidation nor maintenance therapy, remain progression free and have sequential negative MRD measurements, but did not meet criteria for sustained MRD negativity due to their measurements being taken less than a year apart.

**Fig. 4:**
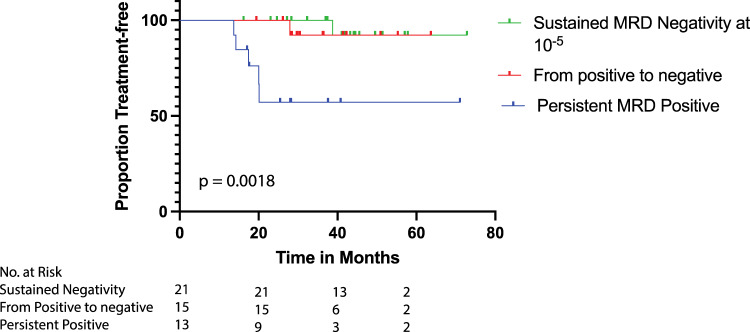
Kaplan Meier curves for time to next treatment (TTNT) according to sequential MRD measurements. According to Sequential MRD Status, with starting point from first negative MRD assessment. Median TTNT was not reached in any group.

IMWG defines sustained MRD negativity as MRD negativity in bone marrow (at a sensitivity of at least 10^−5^) and by PET-CT imaging confirmed minimum 1 year apart. A total of 49 patients, had MRD measurements taken at least 1 year (+/− 15 days) apart, as established by IMWG, and were classified into one of three groups: sustained negativity (*n* = 21, 42.9% of those with two measurements), persistent positive (*n* = 13, 26.5%) and achieved negativity (*n* = 15, 30.6%; were positive at day 100 but attained negativity in subsequent measurement). Among those with two measurements, sustained MRD negativity was associated with longer TTNT in both univariate (HR: 0.07, 95% CI: 0.01–0.63, *p* = 0.02) and multivariable (HR: 0.0075, 95% CI: 0.0002–0.287, *p* = 0.009) models (Fig. 4; Supplementary Table S6). At the time of writing, there were 2 (4.1%) registered deaths in this sub cohort, belonging to patients in the persistent positive group. At cutoff, 48% (10/21) of the patients with sustained MRD negativity have been off treatment for a median duration of 23.5 months. All patients who have suspended therapy have been MRD negative since day 100, but decision to suspend was not influenced solely by achieving sustained MRD negativity, since most patients had discontinued therapy before meeting criteria.

## Discussion

We evaluated the prognostic performance of MRD by NGS to a sensitivity of 10^−6^, its use in combination with functional imaging and the effect of sequential measurements. Our cohort represents a diverse population who underwent induction therapy with a variety of regimens, representing the reality of clinical care. Patients were included regardless of risk category, age, and induction therapy. Although IMWG recommends evaluating MRD for patients who achieve CR or better, this subgroup may represent a lower risk subset of patients overall, making it harder to extrapolate the use of MRD to clinical trials, if not also evaluated in patients with less optimal response to therapy. For this reason, IMWG response was not an exclusion criterion, and our cohort included patients regardless of their IMWG response criteria at time of evaluation.

First, we showed that MRD levels are not associated with cytogenetic risk group nor R-ISS stage. Patients with high-risk genetic subgroups did not have lower rates of MRD negativity, which may indicate that worse outcomes in high-risk categories are not necessarily associated with higher disease burden or ability to respond [27]. We also explored the possibility that specific cytogenetic events were associated with increased disease burden, reflected through higher MRD clone levels, and highlighted that people with 4 or more copies of 1q, have statistically higher levels of the transformed clone. However, the results will need further validation since our cohort included few patients with this cytogenetic event.

Overall, MRD negativity at a sensitivity of 10^−6^ was associated with a longer TTNT, regardless of induction therapy, cytogenetic risk and/or R-ISS stage. The high proportion of relapse patients that had obtained CR or sCR at day 100 further emphasizes the need for more sensitive tests such as MRD. In multivariable models, IMWG response criteria lost significance, and MRD superseded prognostic value. This highlights that the ability to measure deeper response provides better discrimination of patients with superior outcome. The borderline statistical significance in the model may be explained by the lower number of patients achieving negativity with increasing sensitivity thresholds, reducing statistical power. Due to the limited number of registered deaths in our cohort, there were no significant differences between groups, and the only variable associated with decreased survival was R-ISS Stage III. Longer follow up time may reveal significant differences between groups.

Sustained MRD negativity and survival outcomes are not commonly reported. We found that patients who meet criteria for sustained MRD negativity have increased TTNT and OS. At cutoff, there were no deaths reported within this group, and only one documented progression, secondary to a soft tissue plasmacytoma. This is the case even in the three patients with R-ISS Stage III disease and the 5 patients with high-risk genetics, suggesting that sustained MRD negativity may overcome worse prognosis associated with these baseline characteristics, as has been shown by Perrot et al. and Goicoechea et al. [26, 40]. The discontinuation of consolidation or maintenance therapy after achieving sustained negativity remains to be evaluated, but our cohort highlights that the withdrawal of therapy in this subgroup may be a suitable approach, given that patients with sustained negativity who are off treatment remain progression free. Serial MRD measurements can assess the risk of progression in a time-dependent manner and provide a more secure approach for treatment-relate decisions than single MRD measurements.

On the other hand, maintenance and consolidation therapy significantly increase MRD negativity rates, suggesting additional treatment may be appropriate, even in patients with low burden disease detected by NGS MRD assay, given the already apparent effect of achieving (sustained) MRD negativity at 10^−6^ on survival outcomes. Patients who achieved MRD at the end of consolidation/maintenance therapy had very similar outcomes to patients who achieved it at Day 100, emphasizing that regardless at which timepoint of the disease course its reached, MRD negativity is associated with better outcomes.

Long term survivors in our group had decreasing MRD levels overall. Schinke et al. have shown that in lasting survivors, MRD negativity increases over time and remains an important marker for most patients [41]. Only seven patients in our cohort had persistent or increasing MRD levels on sequential measurements, of which three have had progressive disease and two deaths at the time of this writing. One of these 7 patients went from sustained MRD negativity to a positive MRD two years after ASCT, but with no clinical progression to date. However, both previously negative MRD results had disease reported below LOD, and although not apparent in our cohort, may highlight a potential prognostic implication in this subset of patients.

One of the limitations of MRD is the patchy quality of bone marrow, and the possibility of sampling areas not affected by disease. This disadvantage is believed to be resolved by combining MRD evaluation with functional imaging. Given that MRD assessment measures disease at the microscopic level with significantly higher sensitivity than imaging, it was expected that agreement of MRD positivity with PET-CT macroscopic assessment would be poor. Nonetheless, there were two cases where PET-CT was positive in MRD negative patients, but neither of them has had progressive disease. Both patient’s lesions were at sites of previous disease, and given the lack of progression, may represent residual metabolic activity and false positive interpretations rather than disease missed by bone marrow assessment. This emphasizes the need for standardized PET-CT criteria to counteract the lack of interobserver reproducibility. Given that almost all MRD negative patients were also PET-CT negative, the probability of missing disease through NGS MRD assessment is very low, and in this context, the added benefit of performing PET-CT seems small, but additional analysis that re-evaluate the role of PET-CT will be needed to confirm these findings. It is important to determine the MRD false negative rate associated with patchy infiltration to establish the true benefit of performing PET-CT in MRD negative patients.

Nevertheless, a combination of both NGS MRD and PET-CT allows for a comprehensive definition of absence of both macroscopic and microscopic disease. This was evaluated within our cohort, and patients with both negative MRD and negative PET-CT had better progression free survival, compared to those who had either or both positive. Given that the two MRD-negative patients with positive PET-CT remain progression free, outcomes in the “either positive” group are most likely driven by MRD-positive patients. MRD positive patients with positive PET-CT represent a subcohort of patients with higher metabolic rates and may constitute patients with more aggressive disease, thus the inferior TTNT. This group does not solely represent patients achieving inferior IMWG response either, given that 52% (12/23) of patients in the group had ≥CR, and represent 67% (6/9) of relapses within the cohort at time of cut-off. Although PET-CT seems to have limited clinical value in MRD negative patients, it may serve as a tool to differentiate among MRD positive patients, identifying those with more active disease. Combining MRD status and cytogenetic risk was also predictive of TTNT and OS; where both were more favorable in those patients with low-risk genetics who achieve MRD negativity, compared to those who remain positive or have high risk cytogenetics. Paiva et al. suggested that this latter combination could help identify certain patients who obtain CR but should be candidates to more aggressive treatment early in the disease course [18].

To our knowledge this is the first study to incorporate MRD with functional imaging in MM patients who underwent ASCT. Our analysis was performed on a large cohort of patients in a real-life setting, receiving a variety of treatment regimens and showed the benefits of reaching MRD negativity at 10^−6^, but not without some limitations. First and foremost, the retrospective nature of our study prevented us from having predetermined follow up times and close patient monitoring. Furthermore, our relatively limited time of follow up prevented us from identifying important trends in OS which are important for translating TTNT into survival benefit. Due to the lack of a preestablished timepoint for MRD testing in patients not undergoing frontline ASCT, we did not evaluate the role of MRD in patients with multiple lines of therapy prior to ASCT, which could potentially exclude a cohort of patients with higher risk disease where the prognostic value of MRD might differ. Nevertheless, patients were included regardless of their IMWG response at day 100. MRD testing is not standardized nor was it limited to patients achieving ≥CR, but we cannot exclude the possibility of testing criteria variability between different providers.

In summary, our analysis demonstrates that the ability to measure deeper responses provides an opportunity to discriminate a subpopulation of patients with superior outcome. Reaching MRD negativity at 10^−6^ is a strong prognostic factor, even when compared to patients who reach negativity at lower thresholds. These results add to the growing evidence for using MRD to improve the IMWG definition of complete response and its role as a strong prognostic marker for clinical trials. Persistent negativity seems to predict better TTNT and OS, may overcome high-risk features and raises important questions regarding MRD driven therapy. Finally, it seems that there is little advantage of performing PET-CT in MRD-negative patients, given that PET-positivity is rare in this group and outcomes appear to be more dependent on MRD. Additional prospective studies are needed to establish the optimal timing of MRD assessment, the role of combining different types of assessment and further establishing the benefit of obtaining persistent MRD negativity.

## Supplementary information

Supplementary Material

## Data Availability

The dataset generated during and/or analyzed during the current study are available from the corresponding author on reasonable request.
