# Correction: Integrated analysis of next generation sequencing minimal residual disease (MRD) and PET scan in transplant eligible myeloma patients

**DOI:** 10.1038/s41408-023-00856-0

**Published:** 2023-05-26

**Authors:** Rodrigo Fonseca, Mariano Arribas, Julia E. Wiedmeier-Nutor, Yael N. Kusne, Miguel González Vélez, Heidi E. Kosiorek, Richard (Duke) J. Butterfield, Ilan R. Kirsch, Joseph R. Mikhael, A. Keith Stewart, Craig Reeder, Jeremy Larsen, P. Leif Bergsagel, Rafael Fonseca

**Affiliations:** 1 Division of Hematology and Medical Oncology, Mayo Clinic, Phoenix, AZ USA; 2 Dignity Health Cancer Institute, Phoenix, AZ USA; 3 Department of Health Sciences Research, Mayo Clinic, Scottsdale, AZ USA; 4 Translational Medicine, Adaptive Biotechnologies, Seattle, WA USA; 5 Translational Genomics Research Institute, City of Hope Cancer Center, Phoenix, AZ USA; 6 Princess Margaret Cancer Centre, University Health Network, Toronto, ON Canada

**Keywords:** Translational research, Myeloma, Myeloma

Correction to: *Blood Cancer Journal* 10.1038/s41408-023-00794-x, published online 06 March 2023

Following the publication of this article the authors noted and error in Figure 4, “Kaplan Meier curves for time to next treatment (TTNT) according to sequential MRD measurements”. The figure should have displayed only patients who had MRD taken at least 1 year apart (as defined by IMWG), but instead it incorrectly included an additional 8 patients with sequential measurements taken only at least 6 months apart.

The correct figure shows results based on the first MRD taken at least 1 year apart following day 100 evaluation, and should have appeared as below:
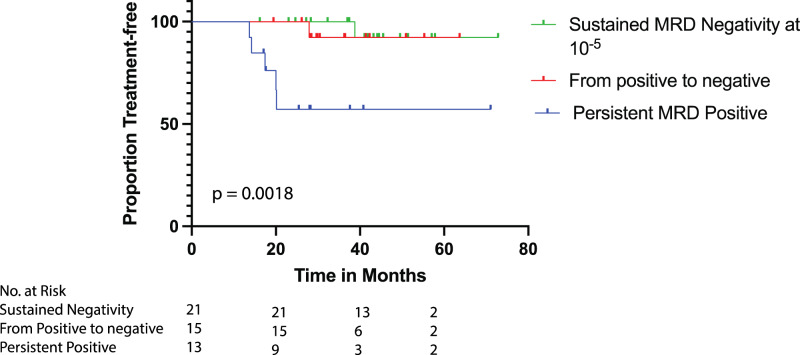


The original article has been corrected.

