# Supplementary material for: Integrated analysis of next generation sequencing minimal residual disease (MRD) and PET scan in transplant eligible myeloma patients
Source: Blood Cancer J. 2023 Mar 6;13(1):32. doi: 10.1038/s41408-023-00794-x (PMC9988896; doi:10.1038/s41408-023-00794-x)
Supplement: Supplementary file 1 — Supplementary Material [file 41408_2023_794_MOESM1_ESM.pdf]

Figure S1. Patient Selection and Status Flowchart

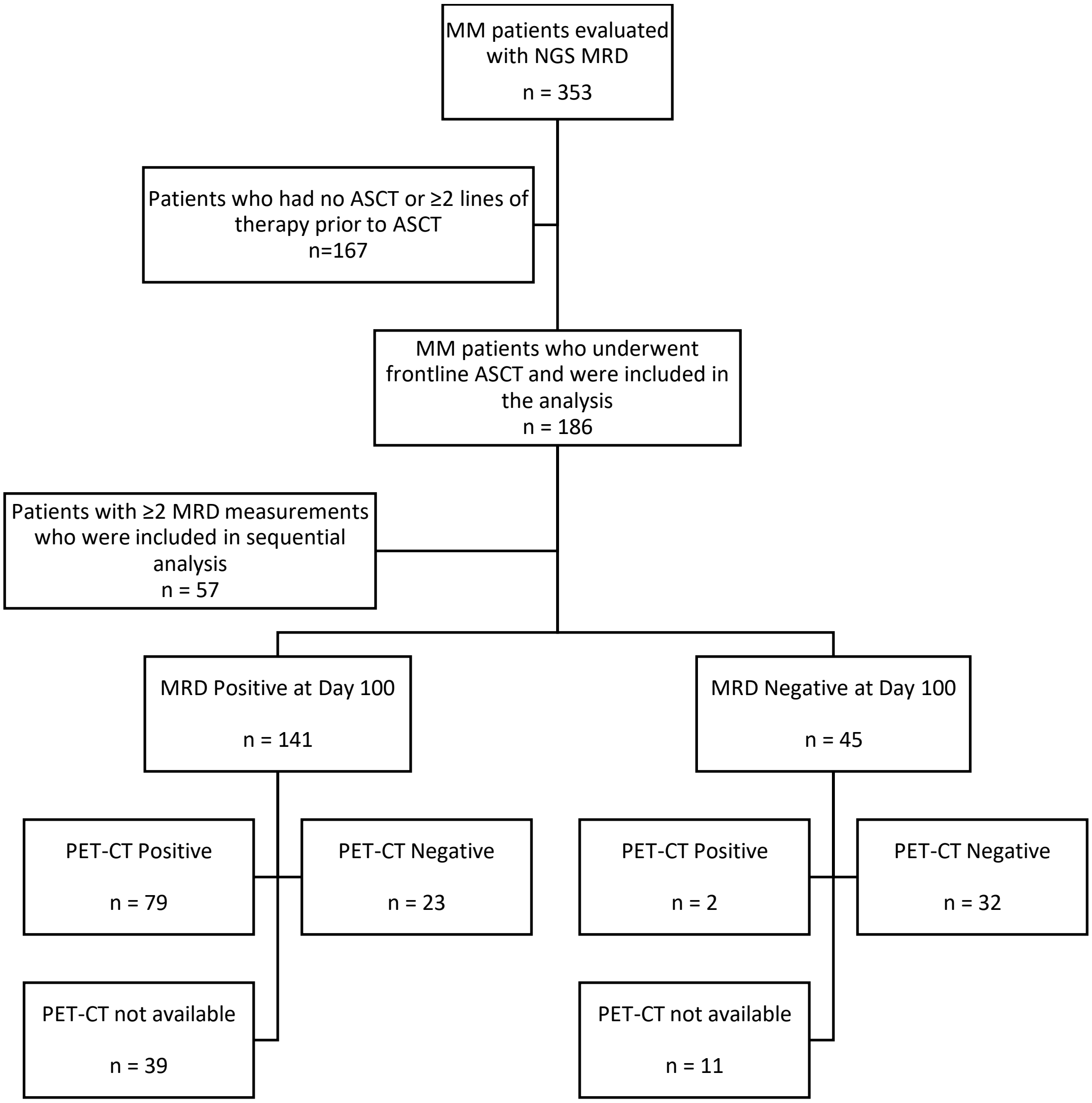

Abbreviations: MM, multiple myeloma; NGS, Next Generation Sequencing; MRD, minimal residual disease; ASCT, autologous stem cell transplant; PET-CT, Positron Emission Tomography and Computed Tomography

Figure S2. Kaplan-Meier Curves for Time to Next Treatment (A) and Overall Survival (B) According to MRD Status in Patients Who Achieved True Negativity Versus Patients Who Achieved Negativity Below Level of Detection (LOD) or Below Level of Quantification (LOQ)

A) Time to Next Treatment

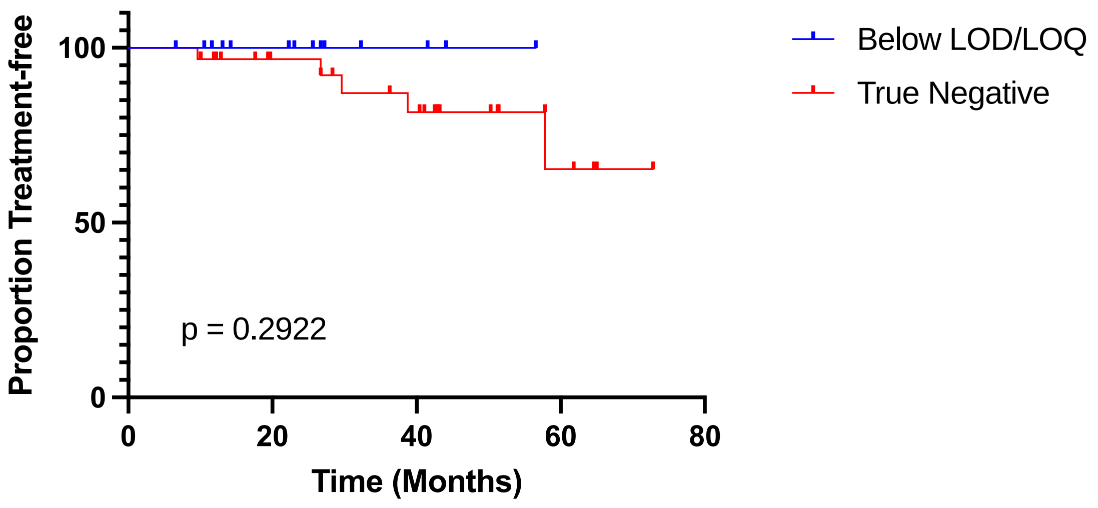

B) Overall Survival

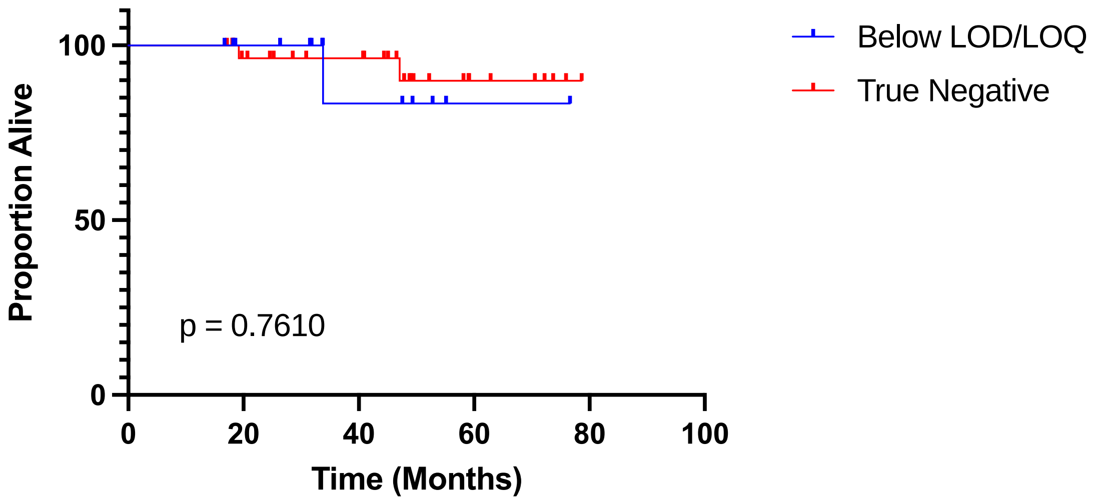

Figure S3. Kaplan-Meier Curves for Progression Free Survival (A) and Overall Survival (B) according to MRD status using sensitivity of 10^{-6}

A) Progression Free Survival

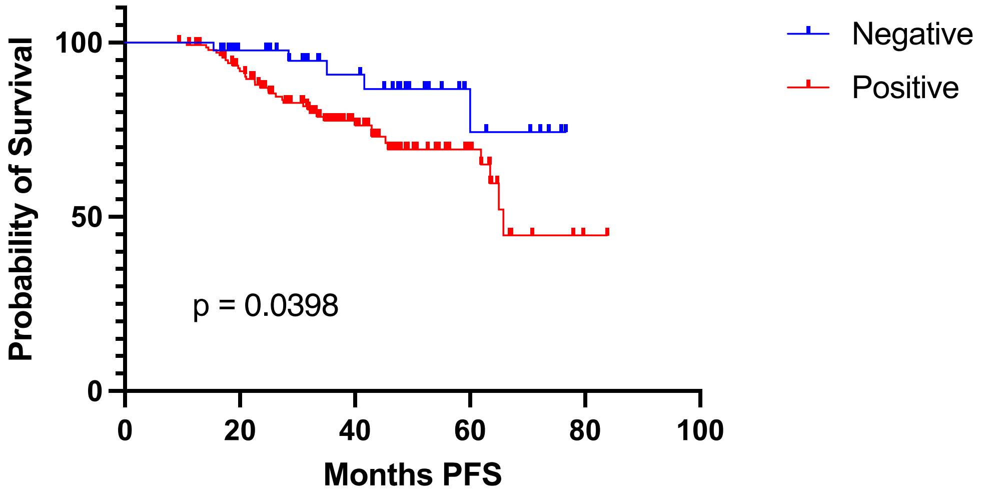

B) Overall Survival

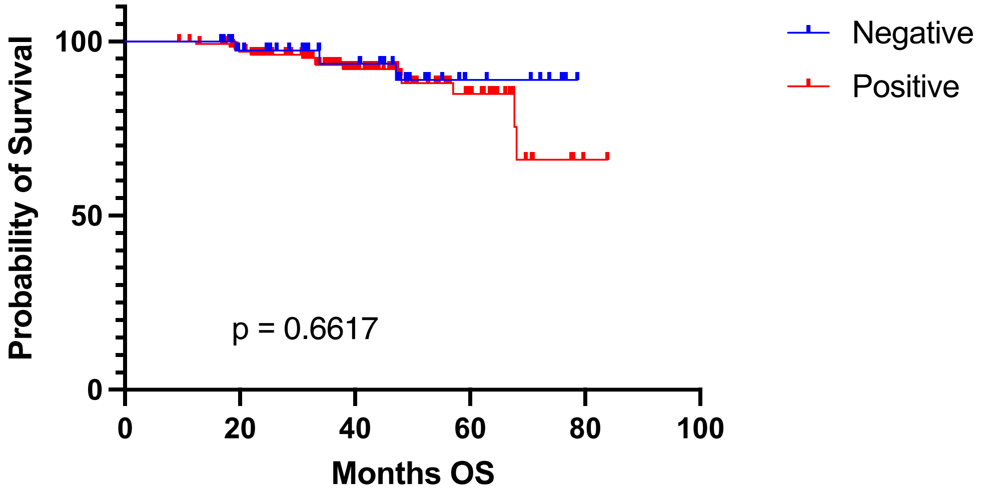

Figure S4. Kaplan-Meier Curves for Time to Next Treatment (A) and Overall Survival (B) according to Revised Multiple Myeloma International Staging System (R-ISS)

A) Time to Next Treatment

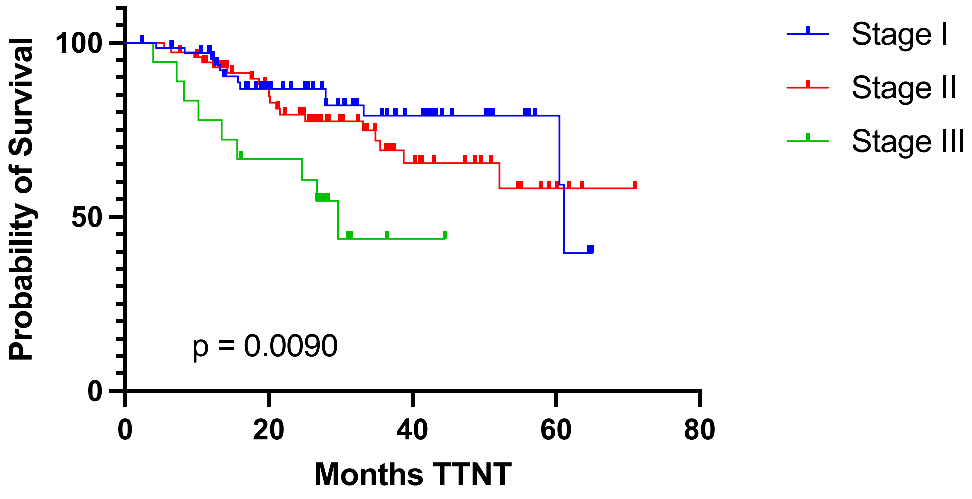

B) Overall Survival

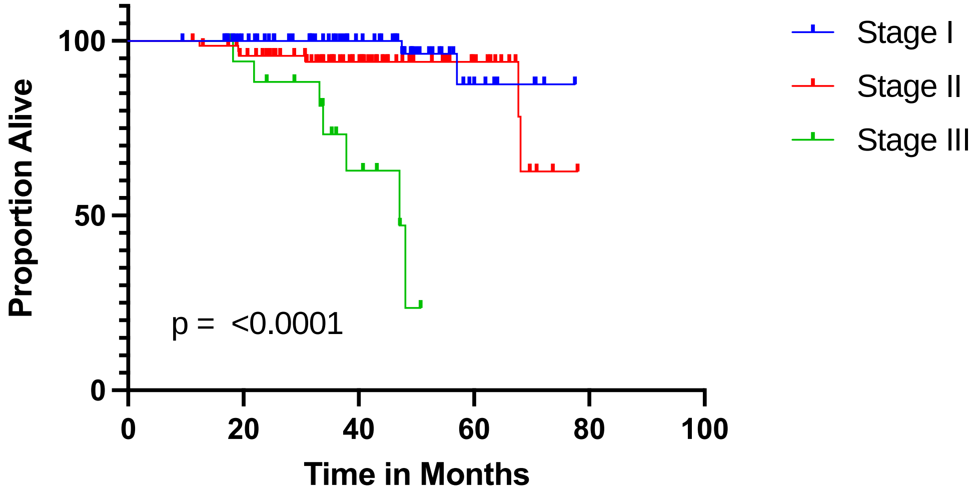

Table S1. Univariate and Multivariable Cox Proportional Hazard Model for Time to Next Treatment (A) and Overall Survival (B) In Patients achieving Complete Response (CR) or Better at Day 100 Post-Autologous Stem Cell Transplant (ASCT)

		**Time to Next Treatment (TTNT)**				**Overall Survival (OS)**			
		Univariate		Multivariable		Univariate		Multivariable	
Variable	Level	HR (95% CI)	P-Value	HR (95%)	P-value	HR (95% CI)	P-Value	HR (95%)	P-value
MRD negativity sensitivity 10^{-6}	Negative vs. Positive	**0.29 (0.1-0.86)**	**0.025***	0.29 (0.085-1.008)	0.051	0.42 (0.09-1.94)	0.2649	0.81 (0.15-4.21)	0.80
Genetic Risk	High Risk vs. Low Risk	**2.87 (1.33-6.19)**	**0.0073***	2.35 (0.87-6.29)	0.09	**4.67 (1.39-15.68)**	**0.0126***	0.79 (0.16-3.98)	0.78
R-ISS Stage	Stage II vs. Stage I	2.94 (0.96-9.05)	0.06	1.98 (0.59-6.60)	0.26	2.5 (0.22-27.87)	0.4556	2.38 (0.20-27.9)	0.49
	Stage III vs. Stage I	**6.87 (1.79-26.3)**	**0.0049***	3.47 (0.74-16.21)	0.11	**93.71 (6-1464.3)**	**0.0012***	**97.01 (5.59-1683.05)**	**0.0017***

* Statistically significant p value

Table S2. MRD Negativity Rates at Day 100 According to Age, Gender, Revised International Staging System Stage, Genetic Risk or IMWG Response Category at Day 100

Variable Summaries	MRD Status at 10^{-6}		p value
	Negative (N=45)	Positive (N=141)	
AGE			0.80 [2]
- Mean (SD)	60.778 (8.301)	60.404 (8.891)	
- Median (Q1, Q3)	62.000 (56.000, 66.000)	63.000 (55.000, 67.000)	
GENDER			0.41 [1]
- Female	18 (40.0%)	47 (33.3%)	
- Male	27 (60.0%)	94 (66.7%)	
R-ISS STAGE			0.91 [1]
- N-Miss	8	19	
- Stage I	17 (45.9%)	51 (41.8%)	
- Stage II	16 (43.2%)	57 (46.7%)	
- Stage III	4 (10.8%)	14 (11.5%)	
HIGH RISK PLUS GENETICS			0.16 [1]
- N-Miss	3	8	
- No	37 (88.1%)	104 (78.2%)	
- Yes	5 (11.9%)	29 (21.8%)	
PET CT INTERPRETATION			0.03 [1]
- Negative	32 (94.1%)	79 (77.5%)	
- Positive	2 (5.9%)	23 (22.5%)	
IMWG RESPONSE CRITERIA			< 0.001 [1]
- sCR	40 (88.9%)	61 (43.3%)	
- CR	0 (0.0%)	18 (12.8%)	
- VGPR	5 (11.1%)	43 (30.5%)	
- PR	0 (0.0%)	16 (11.3%)	
- MR	0 (0.0%)	1 (0.7%)	
- PD	0 (0.0%)	2 (1.4%)	

[1] Chi-square p-value; [2] ANOVA p-value

Table S3. Agreement between MRD Sensitivity at 10^{-6} versus PET-CT Interpretation

MRD 10^{-6} Sensitivity	PET-CT Interpretation		
**Frequency<br>Row Pct**	**Negative**	**Positive**	**Total**
**Negative**	32<br>94.12	2<br>5.88	34
**Positive**	79<br>77.45	23<br>22.55	102
**Total**	111	25	136

Table S4. Minimal residual disease status at 10^{-5} sensitivity (A) and 10^{-6} sensitivity (B) after consolidation and/or maintenance therapy at different time cutoffs.

A)

**MRD Status at 10^{-5}**	**Time Points**		
	At 6 months (N=54)	At 12 months (N=45)	At end of treatment (N=21)
Remained positive	22 (40.7%)	15 (33.3%)	7 (33.3%)
Became negative	13 (24.1%)	19 (42.2%)	7 (33.3%)
Remained negative	19 (35.2%)	11 (24.5%)	7 (33.4%)
Became positive	0 (0%)	0 (0%)	0 (0%)

B)

**MRD Status at 10^{-6}**	**Time Points**		
	At 6 months (N=54)	At 12 months (N=45)	At end of treatment (N=21)
Remained positive	29 (53.7%)	20 (44.4%)	9 (42.8%)
Became negative	12 (22.2%)	14 (31.1%)	6 (28.6%)
Remained negative	13 (24.1%)	11 (24.5%)	6 (28.6%)
Became positive	0 (0%)	0 (0%)	0 (0%)

Table S5. Minimal residual disease status at 10^{-5} sensitivity (A) and 10^{-6} sensitivity (B) at Day 100 post-autologous stem cell transplant (ASCT) and after receiving consolidation and/or maintenance.

A1) After receiving at least 6 months of consolidation or maintenance therapy

**MRD Sensitivity 10^{-5}**	**After consolidation/maintenance**			**P Value**
**At day 100**	Negative	Positive	*Overall*	
Negative	19	0	19 (35.2%)	<0.001
Positive	13	22	35 (64.8%)	
Overall	32 (59.3%)	22 (40.7%)	54	

A2) After receiving at least 12 months of consolidation or maintenance therapy

**MRD Sensitivity 10^{-5}**	**After consolidation/maintenance**			**P Value**
**At day 100**	Negative	Positive	*Overall*	
Negative	11	0	11 (24.5%)	<0.001
Positive	19	15	34 (75.5%)	
Overall	30 (66.7%)	15 (33.3%)	45	

A3) At the end of consolidation and maintenance therapy

**MRD Sensitivity 10^{-5}**	**After consolidation/maintenance**			**P Value**
**At day 100**	Negative	Positive	*Overall*	
Negative	7	0	7 (33.3%)	0.02334
Positive	7	7	14 (66.7%)	
Overall	14 (66.7%)	7 (33.3%)	21	

B1) After receiving at least 6 months of consolidation or maintenance therapy

MRD Sensitivity 10^{-6}	After consolidation/maintenance			P Value
**At day 100**	Negative	Positive	*Overall*	
Negative	13	0	13 (24.1%)	**<0.001**
Positive	12	29	41 (75.9%)	
Overall	25 (46.3%)	29	54	

B2) After receiving at least 12 months of consolidation or maintenance therapy

MRD Sensitivity 10^{-6}	After consolidation/maintenance			P Value
**At day 100**	Negative	Positive	*Overall*	
Negative	11	0	11 (24.4%)	**<0.001**
Positive	14	20	34 (75.6%)	
Overall	25 (55.6%)	20 (44.4%)	45	

B3) At the end of consolidation and maintenance therapy

MRD Sensitivity 10^{-6}	After consolidation/maintenance			P Value
**At day 100**	Negative	Positive	*Overall*	
Negative	6	0	6 (28.6%)	**0.041**
Positive	6	9	15 (71.4%)	
Overall	12 (57.1%)	9 (42.9%)	21	

Figure S5S. Kaplan-Meier Curves for Time to Next Treatment and Overall Survival According to Minimal Residual Disease (MRD) Status at 10-5 (A) and 10-6 (B) Sensitivity Threshold After Consolidation and/or Maintenance Therapy

A1) Kaplan-Meier Curve for Time to Next Treatment According to MRD Status at 10^{-5} Sensitivity After At Least 6 Months of Consolidation and/or Maintenance Therapy

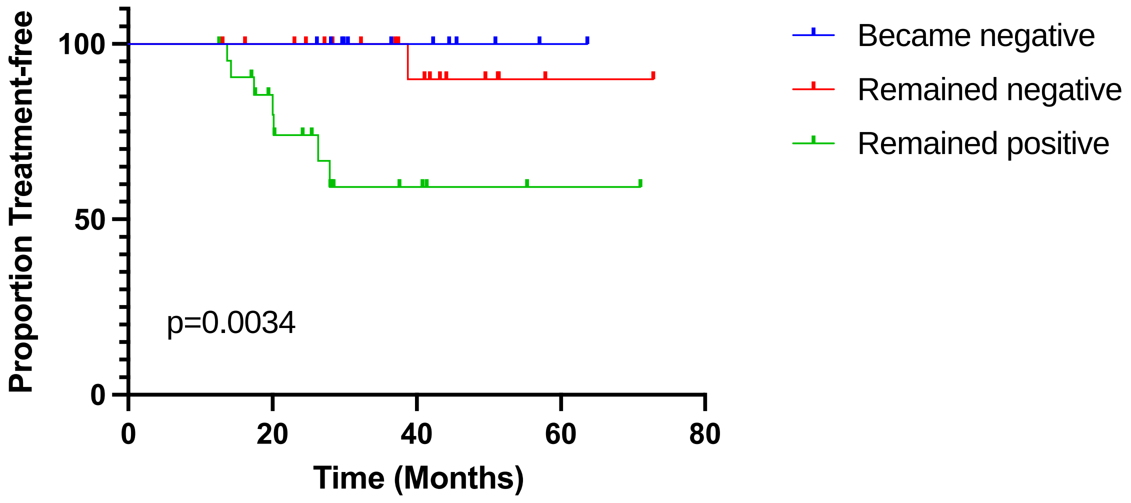

A2) Kaplan-Meier Curve for Time to Next Treatment According to MRD Status at 10^{-5} Sensitivity After At Least 12 Months of Consolidation and/or Maintenance Therapy

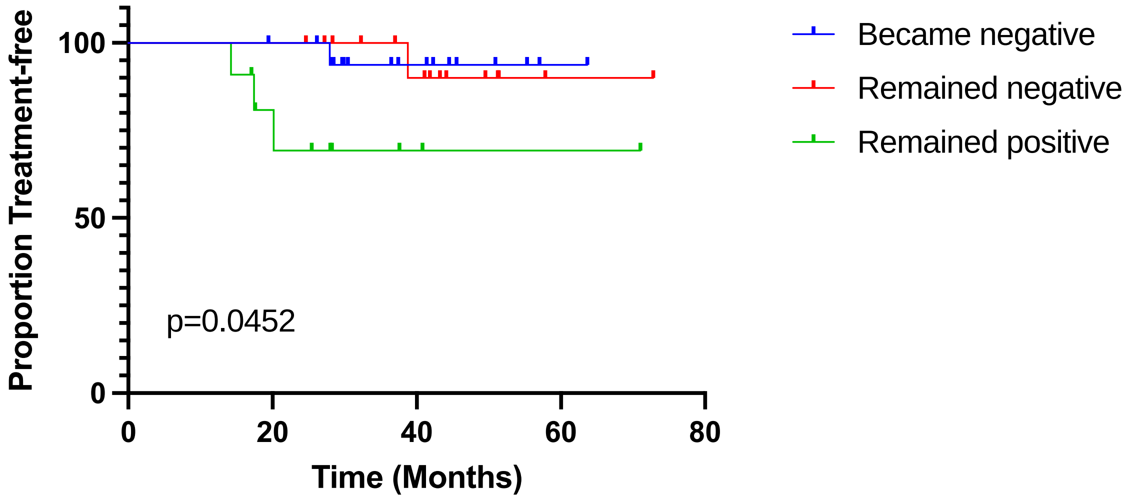

A3) Kaplan-Meier Curve for Time to Next Treatment According to MRD Status at 10^{-5} Sensitivity at End of Consolidation and/or Maintenance Treatment

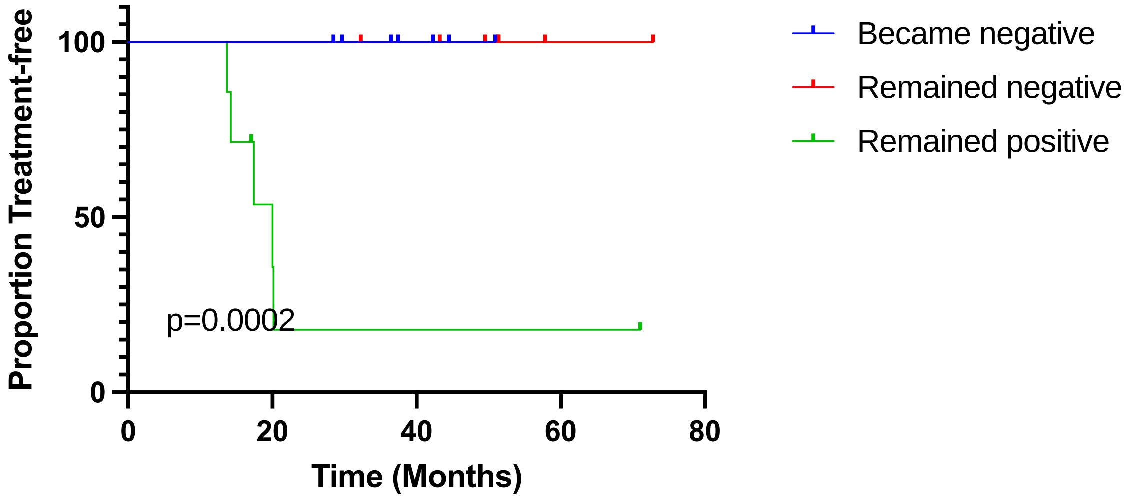

B1) Kaplan-Meier Curve for Time to Next Treatment According to MRD Status at 10^{-6} Sensitivity After At Least 6 Months of Consolidation and/or Maintenance Therapy

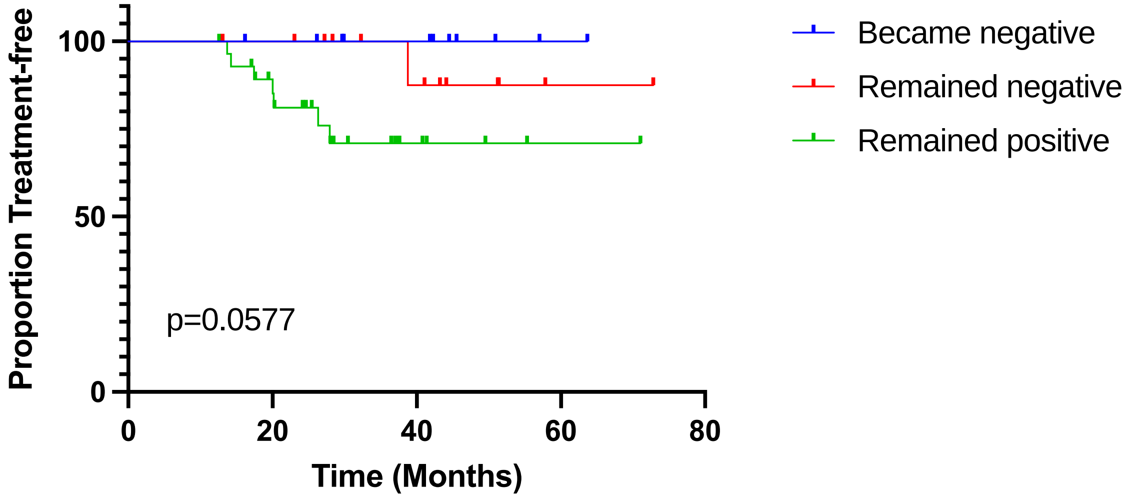

B2) Kaplan-Meier Curve for Time to Next Treatment According to MRD Status at 10^{-6} Sensitivity After At Least 12 Months of Consolidation and/or Maintenance Therapy

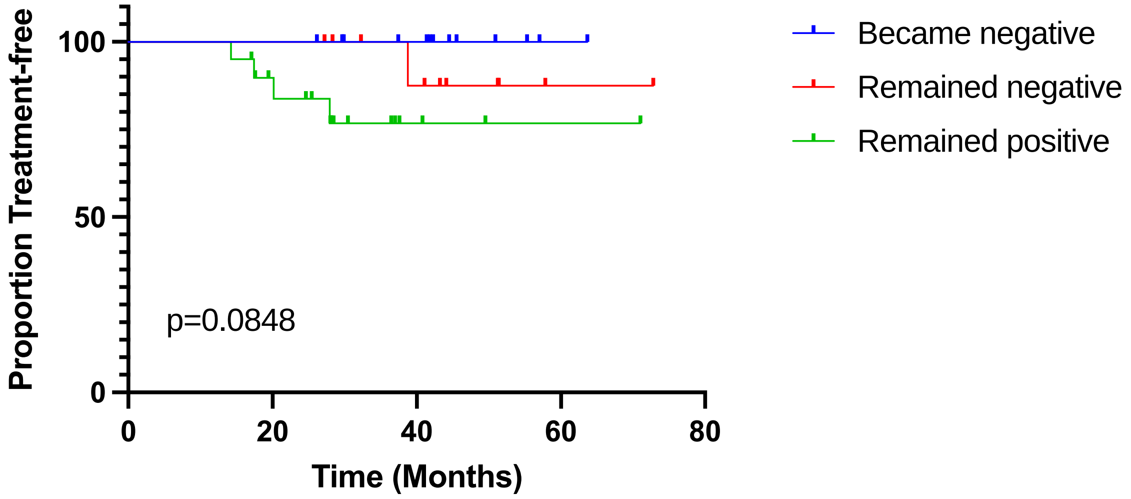

B3) Kaplan-Meier Curve for Time to Next Treatment According to MRD Status at 10^{-6} Sensitivity at End of Consolidation and/or Maintenance Treatment

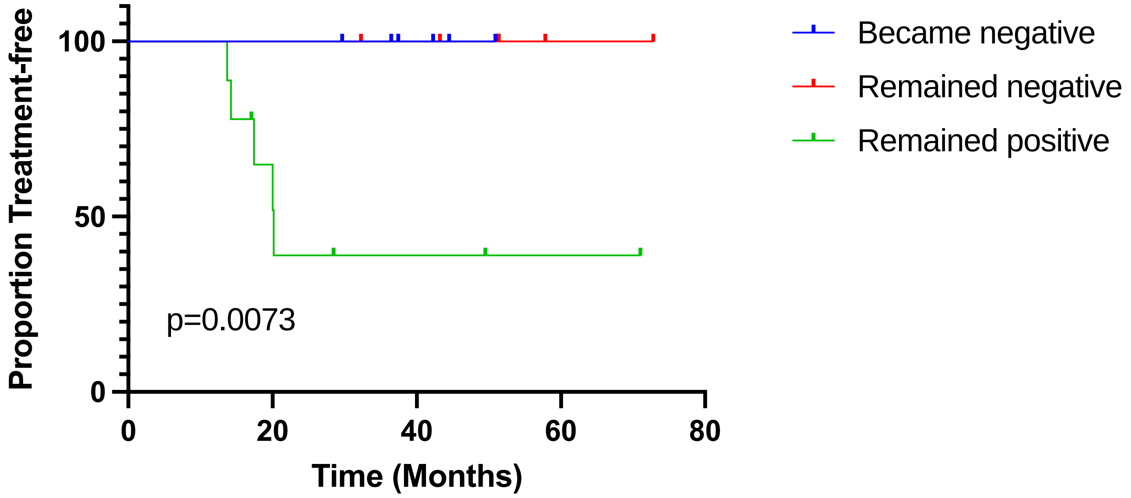

Table S6. Univariate and Multivariate Cox Proportional Hazard Model for Time to Next Treatment (A) in Patients with Two Minimal Residual Disease (MRD) Assays Taken At Least 1 Year Apart

		**Time to Next Treatment (TTNT)**			
		Univariate		Multivariable	
Variable	Level	HR (95% CI)	P-Value	HR (95%)	P-value
MRD negativity sensitivity 10^{-5}	Achieved Negativity vs. Persistent Positive	**0.11 (0.01-0.98)**	**0.0478***	**0.0209 (0.001-0.58)**	**0.023***
	Sustained Negativity vs. Persistent Positive	**0.07 (0.01-0.63)**	**0.0178***	**0.0075 (0.001-0.287)**	**0.009***
Genetic Risk	High Risk vs. Low Risk	0.73 (0.09-6.09)	0.7729	0.52 (0.05-5.93)	0.60
IMWG Response	CR vs. Non-CR	0.33 (0.07-1.51)	0.155	5.77 (0.44-75.6)	0.18
R-ISS Stage	Stage II vs. Stage I	2.09 (0.4-10.77)	0.3792	4.7762 (0.47-48.2)	0.18
	Stage III vs. Stage I	0 (0-inf)	0.9987	0 (0-inf)	0.99
Age		0.95 (0.89-1.03)	0.201	0.8829 (0.78-1.001)	0.05

* Statistically significant p value
